# Rooted in chemistry: 2 secoiridoid glucosides promoting fungal symbiosis

**DOI:** 10.1093/plphys/kiad501

**Published:** 2023-09-14

**Authors:** Henryk Straube

**Affiliations:** Assistant Features Editor, Plant Physiology, American Society of Plant Biologists; Faculty of Science, Department of Plant and Environmental Sciences, Section for Plant Biochemistry, University of Copenhagen, 1871 Frederiksberg C, Copenhagen, Denmark

The intricate relationship between plants and their root microbiome plays a pivotal role in both plant health and their ability to adapt to changing environments. Recent studies have demonstrated the microbiome's role in nutrient uptake, enhancing root architecture, and safeguarding the host against abiotic and biotic stresses (Bakker et al. 2018). Among the many symbiotic interactions between terrestrial plants and microbes, the most prevalent involves arbuscular mycorrhiza fungi (AMF) (Luginbuehl and Oldroyd 2017; Brundrett and Tedersoo 2018). Plants secrete hormones and specialized metabolites into the close soil area around root surfaces called the rhizosphere, influencing the microbiome. New research uncovered that different classes of secreted specialized metabolites, such as alkaloids, saponins, and triterpenes, can influence the microbiome in a positive way for plant fitness (Fujimatsu et al. 2020; Nakayasu et al. 2021; Zhong et al. 2022).

The interaction between AMF and plants is coordinated by signaling molecules. A class of phytohormones called strigolactones (SLs) are considered the most important signaling molecules secreted by the plant to attract beneficial AMF (Luginbuehl and Oldroyd 2017). Other phytohormones also influence AMF symbiosis (Gutjahr 2014). Gibberellin (GA), for example, negatively influences AMF colonization in model plants and decreases the formation of highly branched hyphal structures, called arbuscules (Takeda et al. 2015). These arbuscules are essential for effective nutrient exchange. Surprisingly, researchers discovered that GA treatment of roots from Lisianthus (*Eustoma grandiflorum*; Gentianaceae) promotes colonization by the model AMF *Rhizophagus irregularis* by increasing hyphal branching, although GA treatment significantly suppressed SL production (Tominaga et al. 2021).

In this issue of *Plant Physiology*, Tominaga et al. (2023) identified the involvement of the 2 secoiridoid glucosides gentiopicrin (GPS) and swertiamirin (SWM) in the colonization of Lisianthus by *Rhizophagus* AMF (Fig.).

**Figure.**
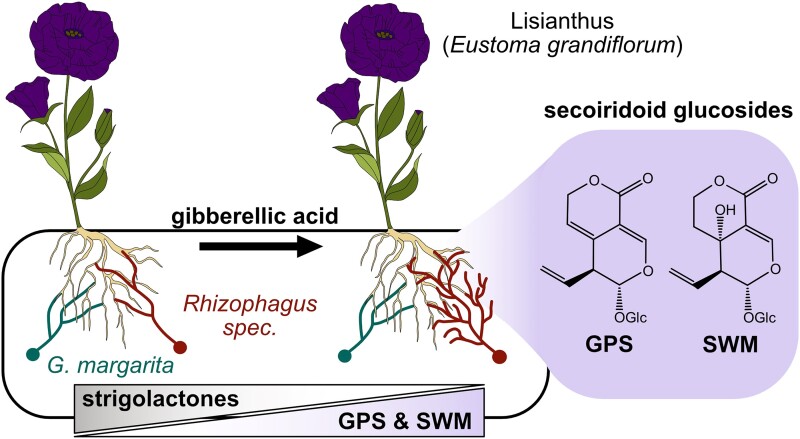
Depiction of the role of the secoiridoid glucosides gentiopicrin (GPS) and swertiamirin (SWM) in AMF symbiosis in Lisianthus. In the presence of gibberellic acid (GA), strigolactone content is decreased, while GPS and SWM abundance is increased. Hyphae of *Rhizophagus irregularis* and *Rhizophagus clarus* (red) are more branched around roots of GA-treated Lisianthus plants, unlike hyphae of the phylogenetically distant AMF *Gigaspora margarita* (green). Structures of GPS and SWM are shown in the purple inset.

Tominaga and colleagues first verified previous results, showing that GA significantly increased the colonization of Lisianthus roots by *R. irregularis* and *Rhizophagus clarus*, supporting the idea that unknown molecules exist stimulating *Rhizophagus* colonization of Lisianthus, although the SL concentration decreased.

The researchers reanalyzed 2 transcriptome datasets of GA-treated Lisianthus roots to identify novel branching factors besides SLs. Intriguingly, they found several genes involved in the secoiridoid pathway to be transcriptionally upregulated in the roots upon GA treatment. As secoiridoid glucosides have not been detected in Lisianthus, the scientists confirmed that the plant is capable of GPS and SWM biosynthesis by identifying these compounds in its roots. It is noteworthy that GPS and SWM were significantly more abundant in roots treated with GA.

To confirm that the compounds are the sought-after branching factors, Tominaga and colleagues used an in vitro bioassay. They treated *R. irregularis*, *R. clarus*, and another AMF, *Gigaspora margarita*, with GPS and SWM. GPS and SWM promoted hyphal branching in concentrations of 1–100 nm in *R. irregularis* and *R. clarus* with comparable activities to a synthetic SL. Hyphal branching was not triggered in *G. margarita* at any concentration, suggesting that GPS and SWM specifically activate *Rhizophagus* hyphal branching.

Using RNAseq, the authors researched the underlying mechanism of secoiridoid glucoside-mediated hyphal branching, analyzing the transcriptome of germinated *R. irregularis* spores treated with 100 nM GPS or a synthetic SL. Both treatments caused comparable transcriptional changes in *R. irregularis,* with transcripts of genes associated with cytoskeletal functions being upregulated. This is coherent with previous work on the role of the cytoskeleton in hyphal branching.

Strigolactones are also signaling compounds in parasitic plants. One of these is *Orobanche minor*, which germinates upon exogenous SL exposure. Neither GPS nor SWM caused *O. minor* to germinate, showing the potential of the secoiridoid glucosides activating AMF symbiosis without affecting the growth of root parasitic plants.

Most exciting, the researchers demonstrated that the external application of GPS to chive (*Allium schoenoprasum*) promoted the colonization of *R. irregularis*, while not affecting plant growth negatively. Note that chive itself does not naturally synthesize secoiridoid glucosides.

β-Glucosidases that potentially hydrolyze secoiridoid glucosides are widely distributed among plants, fungi, and insects. The toxic aglycones of secoiridoid glucosides have been shown to covalently bind to nucleic acids and proteins. A fascinating hypothesis by the authors is that the positive effects of the usually antimicrobial secoiridoid glucosides on *Rhizophagus* AMF are a result of a lack of glucosidases that hydrolyze GPS and SWM in these fungi. Many obligate biotrophic fungi have lost hydrolase genes, including the ones for β-glucosidases. Taken together with the nondestructive infection of AMF, secoiridoid glucosides could be stable during AMF symbiosis, acting as signaling molecules, while maintaining their function as defense compounds against pathogens (Tominaga et al. 2023). This could also explain the missing hyphal branching response of *G. margarita*, as some fungi of the genus *Gigaspora* behave as parasitic.

In conclusion, Tominaga et al. (2023) identified GPS and SWM in Lisianthus, 2 secoiridoid glucosides promoting the colonization of plants by *Rhizophagus* AMF. In the future, it will be most exciting to see the broader impact of the exogenous application of secoiridoid glucosides on the agronomical traits of host plants, like nutrient uptake or increased biotic/abiotic stress resistance. If positive, one could envision engineering the secoiridoid pathway into crop plants to promote their colonization with beneficial AMF.

## Dive Curated Terms

The following phenotypic, genotypic, and functional terms are of significance to the work described in this paper:

gibberellin CHEBI: CHEBI:24250
